# Morphological Characterization of the Polyflux 210H Hemodialysis Filter Pores

**DOI:** 10.1155/2012/304135

**Published:** 2012-11-06

**Authors:** A. Hedayat, J. Szpunar, N. A. P. Kiran Kumar, R. Peace, H. Elmoselhi, A. Shoker

**Affiliations:** ^1^College of Dentistry, University of Saskatchewan, 105 Wiggins Road, Saskatoon, SK, Canada S7N 5E4; ^2^Department of Mechanical Engineering, University of Saskatchewan, 57 Campus Drive, Saskatoon, SK, Canada S7N 5A9; ^3^Saskatchewan Transplant Program, St. Paul's Hospital, 1702, 20th Street West, Saskatoon, SK, Canada S7M 0Z9; ^4^Division of Nephrology, Department of Medicine, University of Saskatchewan, 103 Hospital Drive, Saskatoon, SK, Canada S7N 0W8

## Abstract

*Background*. Morphological characterization of hemodialysis membranes is necessary to improve pore design. *Aim*. To delineate membrane pore structure of a high flux filter, Polyflux 210H. *Methods*. We used a Joel JSM-6010LV scanning electron microscope (SEM) and a SU6600 Hitachi field emission scanning electron microscope (FESEM) to characterize the pore and fiber morphology. The maximal diameters of selected uremic toxins were calculated using the macromolecular modeling Crystallographic Object-Oriented Toolkit (COOT) software. *Results*. The mean pore densities on the outermost and innermost surfaces of the membrane were 36.81% and 5.45%, respectively. The membrane exhibited a tortuous structure with poor connection between the inner and outer pores. The aperture's width in the inner surface ranged between 34 and 45 nm, which is 8.76–11.60 times larger than the estimated maximum diameter of *β* 2-microglobulin (3.88 nm). *Conclusion*. The results suggest that the diameter size of inner pore apertures is not a limiting factor to middle molecules clearance, the extremely diminished density is. Increasing inner pore density and improving channel structure are strategies to improve clearance of middle molecules.

## 1. Introduction

Current data on membrane morphology [1] and patient morbidity and mortality [2] support the need to improve membrane design to better increase the clearance of uremic toxins, particularly the middle molecules [3–8]. As such, more relevant information on current membrane properties and their potential limitations is essential. 

Polyflux 210H is a popular capillary hemodialysis filter produced by Gambro Dialysatoren GmbH, Hechingen, Germany. It is made up of 12,000 Polyamix fibers, with an overall surface area of 2.1 m^2^. Polyamix is a polyarylethersulfone polyvinylpyrrolidone polyamide blend. Each fiber has a reported inner diameter of 215 *μ*m and a wall thickness of 50 *μ*m. The capillary wall of Polyflux filters consists of three layers that are morphologically different [http://www.gambro.com/PageFiles/7431/HCEN2489_5%20Polyflux_H.pdf?epslanguage=en]. An innermost skin layer in contact with the blood that is responsible for solute sieving, thus selecting which solutes be retained or pass through. Bracing the skin layer is a spongy layer that gradually increases in pore size diameter for enhanced diffusive properties. And finally, a finger-form structure that extends to the outer surface of the capillary bestows mechanical strength to the whole architecture [9]. The three layer structure of the capillaries enhances their diffusivity and their sieving characteristics [http://www.gambro.com/PageFiles/7431/HCEN2489_5%20Polyflux_H.pdf?epslanguage=en].

Numerous studies addressed the pore size dispersion in membranes [10–13]. To our knowledge, there have not been independent studies, particularly on pore density, of the Polyflux 210H. We therefore reasoned that the study of this efficient high flux membrane is a step forward to determine what element of membrane structure can be modified. With that in mind, the aim of this study was focused on the structure pore properties of Gambro's Polyflux 210H.

## 2. Materials and Methods

### 2.1. Materials

 The fibers used in this research were fixed using a mixture of 2.5% glutardehyde (SIGMA, G-7651-10ML, Grade I: 50% Aqueous Solution) and 2% paraformaldehyde (EMD Chemicals USA, PX0055-3, 500 g, lot. 49183930) in a 0.1 M phosphate buffer (Mediatech, Inc. Manassas, VA 20109, DPBS, 1X, Sterile). We used a series of mixtures of pure ethanol and distilled water in different ratios to dehydrate the fibers. 

### 2.2. Methods

#### 2.2.1. Apparatus

We cut the plastic cover of the dialyzer using a clamp and handsaw. We used a surgical scalpel to dissect the capillaries longitudinally for characterization of its internal surface that is in contact with the blood.

We used an Ancansco (Toronto, ON, Canada) stereoscopic optical microscope to observe and guide the dissection of the capillaries longitudinally, also, we used an Edwards S150B Sputtercoater to apply a 250 Å layer of gold (purity 99.991%) to render the surfaces of the capillaries conductive for observation under the scanning electron microscope (SEM) and the field emission scanning electron microscope (FESEM). The SEM we used is a Joel JSM-6010LV, and the FESEM is a SU6600 Hitachi.

We estimated the maximum diagonal of selected uremic toxins' molecules using the Crystallographic Object-Oriented Toolkit (COOT) software [14].

#### 2.2.2. Procedures

**(a) Cutting the Dialyzer Open and Fiber Extraction**

The cutting was pursued transversely and longitudinally without disturbing the fibers inside. Then, using scissors, a bunch of fibers were cut from an area remote from that cut by saw to ensure that no debris has come in contact with the fibers.

**(b) Fixation of the Polyflux 210H Fibers**

Fixation was pursued in a Petri dish using a mixture of 2.5% glutardehyde and 2% paraformaldehyde in a 0.1 M phosphate buffer, and left for fixation overnight at room temperature. The fixed fibers were then washed three times in the same phosphate buffer, ten minutes each. Following the final rinse, the fixed fibers were dehydrated in a series of ethanol/distilled water mixtures (50% ethanol, 70% ethanol, 90% ethanol) for 20 minutes each, followed by dehydration twice in 100% ethanol, for 20 minutes each. The fibers were then left to dry in air.

**(c) Dissection of the Dialyzer Capillaries**

To examine the internal surface (skin layer) of the fibers, some fibers were placed on a carbon double sided adhesive scanning electron microscopy (SEM) tape glued from the other side to an SEM stub. The SEM stub, with the fibers glued to its surface, was transferred for viewing under the stereoscopic optical microscope. The fiber walls were dissected longitudinally using a surgical scalpel while being examined under a magnification of 25x.

**(d) Coating the Capillaries**

For better imaging of the capillaries, we coated all capillaries including the dissected ones with gold. The gold, conductive coating of the substrates, was applied for 4 minutes at 7 mbar (700 Pa). These coating parameters yielded a 250 Å layer of gold on the exposed surfaces of the capillaries.

**(e) SEM and FESEM Observation of the Capillaries**

Both the SEM and the FESEM were used to characterize the morphologies and apertures on the capillaries from the inner and outer surfaces, as well as the cross-sectional morphology and its tortuous structure. They were used to quantitatively measure the sizes of apertures as well as the capillaries' outer diameter and wall thickness. The FESEM was advantageous over the SEM at higher magnifications, where the images appeared clearer, and with more detail. Photomicrographs were taken for the different aperture sizes on the inner and outer surfaces of the capillaries for further calculations.

**(f) Measurements of the Hemodialyzer's Capillaries (Fibers)**

Both the outer diameter and wall thickness of the dialyzer's capillaries were measured using the SEM and the FESEM. The measurements were made on two capillaries, with ten measurements each. The average of the capillary's diameter and thickness as well as the standard deviations were calculated.

**(g) Estimation of the Open Areas on the Capillary's Surface**

With the aid of the SEM and the FESEM, we analyzed the photomicrographs of the inner surface (skin layer) of the capillary that is in contact with the blood, as well as the outer surface (finger layer) that is in contact with the dialysate. We measured the range of pore sizes on the inner and outer surfaces. We printed SEM and FESEM photomicrographs, each of which was 15 × 25 cms. We then divided each photo into 5 × 5 cms areas and calculated the number of pores and size of each. Then, we calculated the cumulative area of pores and divided this area by the overall area, which is 25 cm^2^. This yielded a fraction of open space per area. As such, we got a total of 10 areas of 5 × 5 cms for each of the pores on the inner surface and outer surfaces of the capillaries. The results were then analyzed statistically, yielding the range, mean, and sample standard deviation.

**(h) Measuring the Maximum Molecular Dimension of Selected Uremic Toxins and Albumin**

We obtained the codes for selected protein uremic toxins from the Protein Data Bank and used them in the Crystallographic Object-Oriented Toolkit (COOT) software. We analyzed the molecules one at a time, by rotating the molecule in three dimensions around its axis, and then measured its maximum dimension after taking 10 measurements in random directions. The maximum dimension for each uremic toxin was then recorded. The maximum diagonal of urea and glucose was measured using Spartan software (Wavefun, CA, USA).

## 3. Results

This study is approved by the Ethics Board of the University of Saskatchewan.

### 3.1. Characterization of the Surfaces' Morphology and Open Space Areas of Polyflux 210H

**Figure 1:**
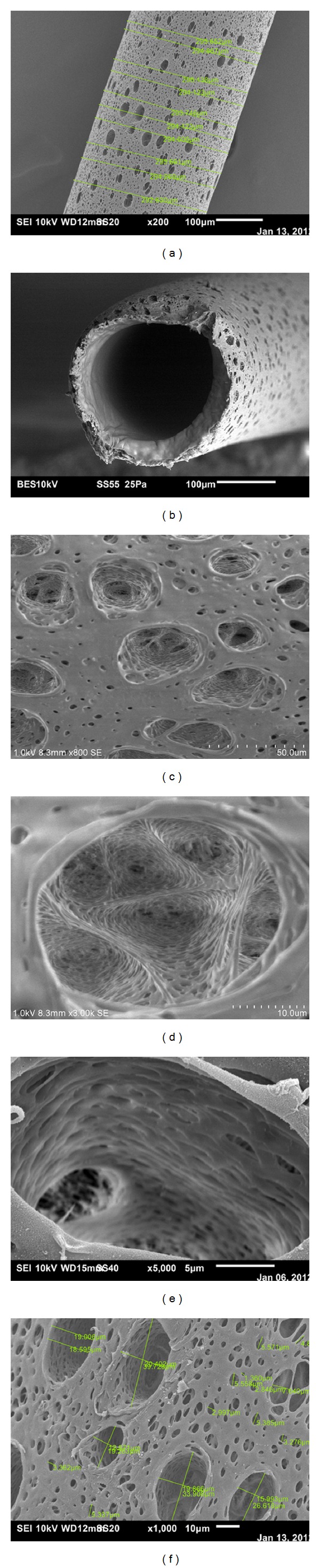
(a) Outer surface of a Polyflux capillary at 200x with measurements, (b) view of the cross section of a Polyflux capillary at 250x, (c) morphology of outside pore in contact with the dialysate at 800x, (d) morphology of outside pore in contact with the dialysate at 3000x, (e) morphology of outside pore in contact with the dialysate at 5000x, and (f) pores on the dialysate side of the Polyflux 210H capillary at 1000x with measurements.

**Figure 2:**
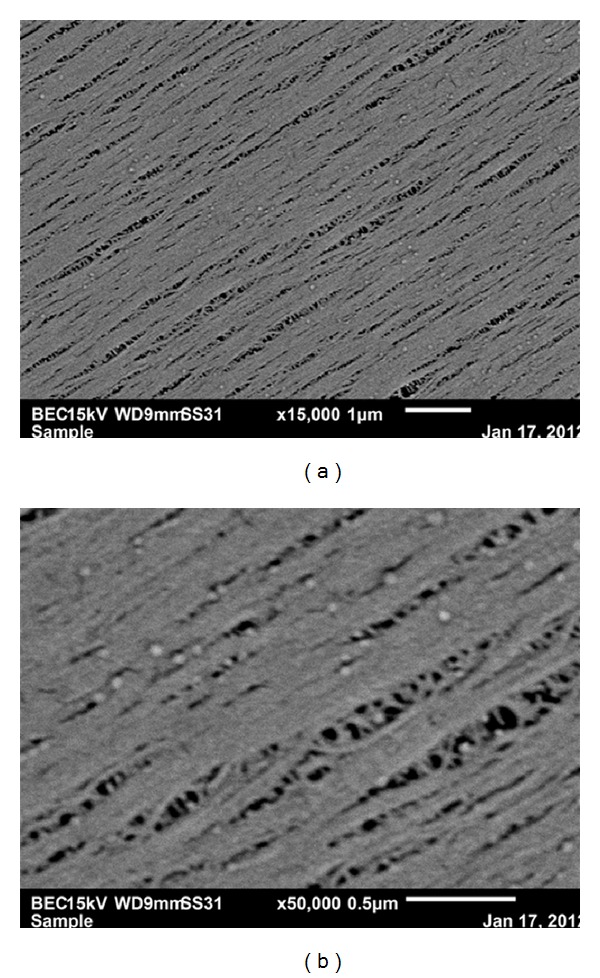
(a) Morphology of the pores in contact with the blood at 15,000x. (b) Morphology of the pores in contact with the blood at 50,000x

Figure 1(a) shows the outer surface of a Polyflux capillary at 200x with measurements, while Figure 1(b) shows the plane view of the capillary's cross-section at 250x. Figures 1(c)–1(e) show the morphologies of the outside pore in contact with the dialysate at different magnifications, and Figure 1(f) shows the pores measurements on the outside surface. The range of pore size in contact with dialysate (width) varied between 0.45 *μ*m and 20.40 *μ*m. The photomicrographs showed how the structure consisted of intricate openings. Figures 2(a) and 2(b) show the morphologies of the pores in contact with the blood at 15,000x and 50,000x, respectively. The morphology of the inner surface of the Polyflux capillary was smoother and compacted in contrast to that on the dialysate side. The openings were much wider in the middle as compared to the tapered ends. The range of pore size in contact with the blood (width) varied between 34 nm and 45 nm.

**Figure 3:**
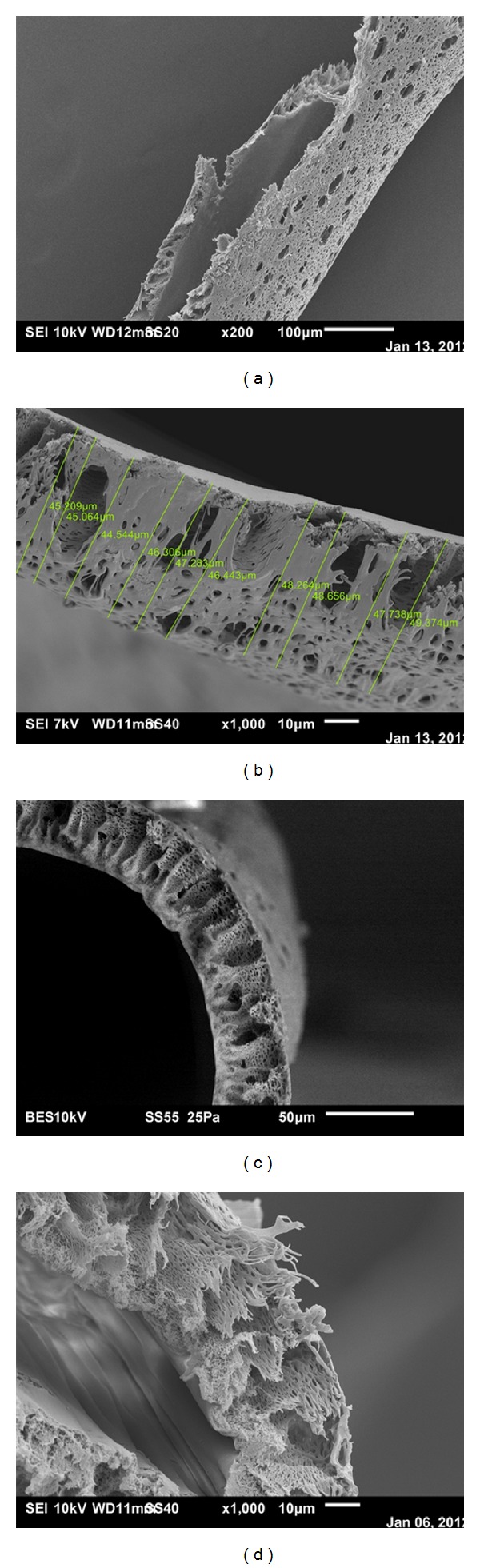
(a) Dissected Polyflux capillary showing the morphologies of the outer and inner surfaces at 200x, (b) capillary wall thickness of Polyflux 210H at 1000x with measurements. (c) Cross section of Polyflux 210H illustrating the radial supporting structure that extends from the inner skin layer of the capillary to the outside surface. (d) Cross section of Polyflux 210H at 1000x illustrating the radial variation in compactness in the capillary's porosity.

Figure 3(a) shows a dissected Polyflux capillary with the morphologies of the outer and inner surfaces. Also, we measured the thickness of the capillary's wall at different points and a recorded a sample of 10 measurements on each capillary examined. We applied this on three different capillaries selected randomly, and then calculated the mean and standard deviation of the measurements on each capillary.  Figure 3(b) shows the capillary wall thickness of Polyflux 210H at 1000x with measurements and Figures 3(c) and 3(d) show the supporting finger structure extending from the skin inner layer to the outer surface.  Table 1 summarizes our results on the primary characteristics of the Polyflux 210H capillaries, while Table 2 lists the measured maximum diameter of selected uremic toxins. 

### 3.2. Maximum Dimensions of Selected Uremic Toxins and Albumin

The maximum diameter of selected uremic toxins is listed in Table 2 as measured by COOT software.

## 4. Discussion

The results raise several points for discussion. First, the pores on the skin layer were more elliptical in shape than round In this study we measured the maximum diameter of *β*2-microglobulin to address whether it can go through the inner pores. The result shows clearly that the *β*2-microglobulin whose maximum dimension is 3.88 nm can clear through the holes on the inner surface of the dialyzer because these pores have a diameter that ranges in size between 34–45 nm. 

 Measurement of molecule's diameter deserves a comment. In one study [15], the diameter of *β*2-microglobulin was estimated to range between 3.14 to 3.18 nm. In another method [16], *β*2-microglobulin's diameter was simply calculated as 3.0 nm using Stoke's equation that relates molecular weight to the radii of molecules, and treats the molecules as spheres. This is a less accurate representation of the *β*2-microglobulin, since the molecule is not spherical. Thus, we reached the first conclusion that the size of the inner pore apertures per se is not a limiting factor for clearance of middle molecules. 

Limited clearance of *β*2-microglobin in clinical practise is then caused by other factors such as shape of pores and molecular geometry [17].

 Another relevant finding is that we failed to see direct communication between the outer and inner surface pores. In fact the orientation of the channels within the fiber walls was random and highly variable and thus adds to limitations to molecule fluxes through the capillary wall.

The results show the striking low density of inner pore apertures. This finding is similar to an earlier study that concluded a pore density of 10% in Hemophan membrane [18]. A recent study of polyethersulfone (PES) [1] demonstrated the unsatisfactory pore shape of the PES membrane. The study did not estimate the pore density, however. Their results were also supported by the findings of other investigators in other hemodialysis membranes [19]. It is significant to refer to a study [20] where they showed a steady increase of porosity from the thin polymer, dense region to the outer surface of the membrane. In total, the results corroborate the general notion that current methods used to produce dialysis membranes resulted in a nonuniform pore size distribution.

 This study has important limitations. The first is that our model takes into account a dry medium. The second limitation is that we did not apply or compare our model to that based on Stoke's radius equation. Stokes equation that relates molecular weights to molecular radii treats molecules as spheres. Instead, we used Crystallographic Object-Oriented Toolkit (COOT) to estimate the largest dimensions of the toxins of interest, which are branched and nonspherical. We believe that our method is more accurate than using Stoke's radius equation.

## 5. Conclusions

Future hemodialysis membrane designs are governed by clearance of uremic toxins, retention of essential proteins, and prevention of backfiltration.The means of accomplishing these objectives can be fulfilled through optimizing pore size and pore density to maximize flux of toxins. Improving outer and inner membrane surface connection and increasing inner pore density seem obvious potential strategic targets to improve hemodialysis membrane efficiency.

## Figures and Tables

**Table 1:** Characterization summary of Polyflux 210H and the results are presented as mean ± standard deviation.

Characteristic	Gambro's Polyflux 210H (Mean ± standard deviation)	Mean absolute percent Error ± standard deviation
Open pore space (in contact with the blood)	5.45% ± 1.41	
Open pore space (in contact with the dialysate)	36.81% ± 14.62	
Outer diameter	294.58 *μ*m ± 1.05	11.16% ± 0.40
Wall thickness	46.88 *μ*m ± 1.65	6.22% ± 3.28
Inner surface pore width range	34 nm–45 nm (40.11 nm ± 3.62)	
Outer surface pore width range	0.45 *μ*m–20.40 *μ*m (11.36 *μ*m ± 7.80)	

**Table 2:** Measured maximum diameter of glucose and selected uremic toxins.

Molecule	Maximum diameter (nm)
Urea	0.48
Glucose	1.0
Endothelin	2.60
*β*2-microglobulin	3.88
Complement factor D	5.12
